# Fostering Need-Supportive Behaviors in Physical Education Teachers and Parents: A Cluster Randomized Controlled Trial Study Protocol of a Web-Based Intervention on Secondary School Students’ Physical Activity

**DOI:** 10.3390/mps5050083

**Published:** 2022-10-18

**Authors:** Pille-Riin Meerits, Henri Tilga, Andre Koka

**Affiliations:** Institute of Sport Sciences and Physiotherapy, University of Tartu, Ujula 4, 51008 Tartu, Estonia

**Keywords:** children, adolescents, self-determination theory, autonomy, competence, relatedness, motivation, trans-contextual model of motivation, theory of planned behavior

## Abstract

Despite various benefits of physical activity, children are increasingly inactive. Both school physical education classes and support from parents are important determinants of physical activity level of children and adolescents. We aim to develop a web-based intervention for physical education teachers and parents to teach them to be more need-supportive towards children when discussing physical activity and thus increase children’s autonomous motivation towards it. The study will adopt a waitlist-control design with cluster randomization by schools. The intervention content is based on self-determination theory. Specifically, the teachers and parents will be introduced to a series of motivation and behavior change techniques to help them satisfy the children’s psychological needs for autonomy, competence, and relatedness in physical activity. The targeted group in the six-week intervention is comprised of students aged 12–14 years. The primary outcome variable, physical activity of students, will be assessed via self-report questionnaires at baseline, post-intervention, one-month and six-month follow-up. Web-based intervention programs are cost-effective, allow self-paced learning and enable reaching larger audiences. If this project proves to be effective, a highly valuable web-based solution would be available for PE teachers and parents to help increase students’ physical activity levels.

## 1. Introduction

Physical activity (PA) has been demonstrated to have several adaptive health outcomes. The benefits in children and adolescents are physical (e.g., cardiometabolic and bone health), psychological (e.g., reduce depressive symptoms), and cognitive (e.g., better academic performance) [1]. The WHO guidelines state that children and adolescents should get an average of 60 min of moderate-to-vigorous physical activity (MVPA) daily [2]. Nevertheless, most children and adolescents in Europe do not meet the guideline levels of PA [3,4,5,6]. For example, a recent study in Estonia demonstrated that only 2.5% of participating 11–15-year-olds met the daily recommended PA level for seven consecutive days [7]. Moreover, levels of PA in this age group tend to decline with age [8,9]. Low PA during childhood and adolescence may increase the risk of developing various chronic diseases in adulthood, contributing to increased morbidity and lower quality of life [10]. Considering the beneficial attributes of PA and detrimental attributes of inactivity, it is crucial to identify the psychological determinants of PA to use as guiding evidence to develop effective interventions to promote PA in children and adolescents.

**Figure 1:**
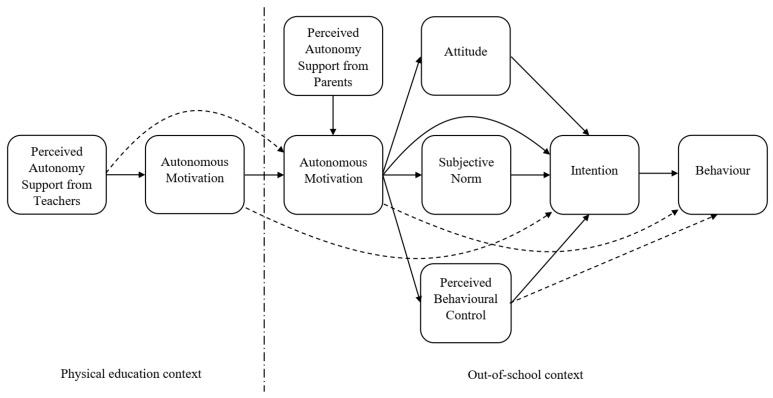
The trans-contextual model of motivation (TCM) expanded by parental autonomy support. Adapted with permission from ref. [24]. 2022 Taylor & Francis.

Physical education (PE) classes in school have been recognized as an existing network through which PA interventions can be delivered to virtually all children [11]. By providing support to the basic psychological needs of autonomy, competence and relatedness, PE teachers can foster students’ autonomous motivation towards PE, but more importantly, also autonomous motivation towards out-of-school PA. The trans-contextual model of motivation (TCM; Figure 1) is a well-known theoretical model developed to identify the determinants of PA within both PE and out-of-school contexts [12]. The TCM is a multi-theory model that integrates constructs and hypotheses from the self-determination theory [13], the theory of planned behavior [14] and the hierarchical model of motivation [15]. To date, research applying the TCM has identified that autonomy-supportive teaching in a PE context and autonomy support from parents are related with higher levels of self-reported PA outside of school among children and adolescents through autonomous motivation in both PE and out-of-school contexts, social cognition beliefs, and intention towards PA [16,17,18].

González-Cutre and colleagues [19] have extended the trans-contextual model of motivation by including additional variables, namely autonomy support from peers and parents. The tested extended TCM supported the main postulates of the model, and the authors further suggest that randomized controlled interventions, including parents and peers, should be designed in the future.

As a recent addition to research based on the self-determination theory, Teixeira, and colleagues [20] have classified 21 techniques that comprise self-determination theory interventions. These motivation and behavior change techniques (MBCTs) are organized by the psychological needs of autonomy, competence, and relatedness. Furthermore, Ahmadi and colleagues [21] have classified 57 teacher motivational behaviors based on the self-determination theory’s psychological needs. The classification clearly describes the possible application and estimates motivational effect of each behavior, making it easier for researchers and teachers to apply the methods.

Numerous intervention studies in which PE teachers have adopted autonomy-supportive techniques have demonstrated to be effective in changing PE-related outcomes for students, such as greater need satisfaction, higher autonomous motivation and engagement, while lowering need frustration and amotivation in lessons [22]. On the other hand, only a few TCM-guided studies have administered interventions in PE context with the aim of changing students’ PA behavior outside of school [23,24,25]. Studies by Barkoukis with colleagues [23] and Schneider with colleagues [24] failed to support the efficacy of the autonomy-supportive intervention for PE teachers in changing out-of-school PA behavior among secondary school students. The authors concluded that one of the main reasons for the null effect may be that the intervention did not target other potentially influential determinants of PA proposed in the TCM, such as autonomy support from parents [26,27]. It was proposed that including additional training components to the intervention with autonomy support from other social agents, such as parents may have resulted in larger effects on out-of-school PA among children and adolescents [24]. Another potential issue raised by the authors was the short and very intensive intervention period that did not allow self-paced learning and assimilation of learned behavior. On the contrary, the study by Sevil-Serrano and colleagues [25], in which both PE teachers and parents were trained to become more need-supportive, proved to be effective in enhancing out-of-school PA levels in secondary school students. However, they did not isolate which components of the intervention were the most effective in bringing about behavior change.

According to Reeve and Cheon [22], technology can be integrated into future need-supportive intervention research to improve educational practice in several ways. Firstly, future interventions could be delivered using an online approach as being more personalized and self-paced. It would also be possible to include additional materials, such as a video presentation of an exemplary autonomy-supportive teacher who provides a voice-over to explain what she was doing and why. To the best of our knowledge, only a few interventions so far have successfully delivered need-supportive components using a web-based format [28,29]. For example, the study by Tilga and colleagues [29] demonstrated that an entirely web-based autonomy-supportive intervention was effective in changing student PE-related outcomes. Moreover, this study showed long-lasting effects in a 15-month follow-up measurement [30]. Secondly, providing autonomy support to adolescents is highly important to support their motivation towards PA, but a challenging task to monitor for a PE teacher or a parent who is doing this for the first time. To help participants develop need-supportive communication, we have an online forum where teachers and parents are invited to discuss their successes and setbacks in practicing the necessary skills and techniques.

To date, there is no empirical evidence on the efficacy of the unique and combined effects of a web-based intervention program designed to train need-supportive behaviors in both PE teachers and parents simultaneously on adolescents’ motivation towards and actual participation in out-of-school PA. We adopt a web-based format for delivering need-supportive interventions to PE teachers and parents that will be augmented with an online forum to maximize the effectiveness of interventions.

The main objective is to develop and test the effectiveness of this combined intervention on Grade 6–7 students’ out-of-school PA. Firstly, we expect to find the main effects of the PE teacher-delivered and parent-delivered components of the need-supportive intervention on Grade 6–7 students’ PA. Secondly, we expect an interaction effect of the teacher- and parent-delivered components of the intervention such that students assigned to receive both the teacher- and parent-delivered components exhibit the greatest increase in PA participation compared to students assigned to receive either component alone and students assigned to the control condition that do not receive either component.

## 2. Experimental Design

### 2.1. Design, Randomization

**Figure 2:**
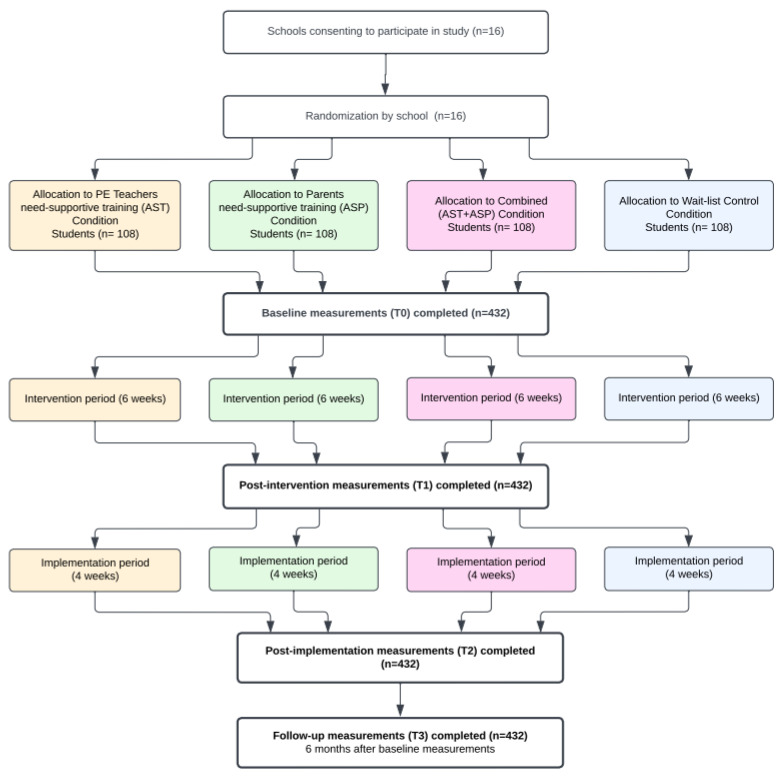
Participant flow diagram.

The study will adopt a 2 (PE teacher delivered intervention component: present/absent) × 2 (parent-delivered intervention component: present/absent) × 4 (time: baseline, post-intervention, one- and six-month follow-up) cluster-randomized waitlist control design with simple 1:1:1:1 randomization by school. Hence, the study will have three experimental groups (teacher-delivered, parent-delivered, and combined teacher- and parent-delivered intervention) and a waitlist control group (Figure 2).

Respective of their school, the PE teachers and parents will be assigned either to participate in a six-week web-based need-supportive intervention program or to continue teaching and parenting as they currently do (i.e., control group). Students are blinded to allocation. The web-based training for teachers and parents will be preceded by pre-trial data collection during which questionnaires containing baseline measures (i.e., demographics, psychological, and behavioral measures) will be administered. The six-week training program will be followed by a one-month implementation period during which the teachers and parents will apply their training in regular PE classes and at home, respectively. The one-month implementation period will be followed by post-trial data collection, as well as one- and six-month follow-up data collections during which questionnaires with identical measures will be administered again. The waitlist control group will receive the web-based need-supportive intervention program after the six-month follow-up data collection.

The study will be registered in ISRCTN registry before enrolment of the first participant. The current stage of the study is ‘not recruiting’. The protocol described here is dated 30 August 2022 and it has been amended once after approval. Primary reason for amendment was to take out references to smartphone application to complement the intervention. Due to the lack of funding, we are going forward without the application.

### 2.2. Sample Size Calculation

Power calculation was conducted to estimate the required sample size. Calculations were conducted using G*Power version 3.1.9.7. We aim to achieve 80% statistical power to detect medium effect sizes when considering four measurements (baseline, post-intervention, one- and six-month follow-up) at the within level (correlated 0.50), four conditions at the between level, and an alpha level of 0.05. The statistical power analysis revealed that the required sample size would be n = 308 (i.e., 77 students allocated to each of the four study groups). However, considering the potential drop-out rate of approximately 40% shown in multiple follow-up studies of PA [31], we aim to recruit 432 students at baseline (i.e., 108 students allocated to each of the four study groups).

### 2.3. Participants

Eligible participants will be Grade 6–7 students without restrictions on their participation in PE classes, their parents and qualified full-time secondary school PE teachers. Assuming there are, on average, two parallel classes of Grade 6 students in each school, and that an average class size of students is 20, we aim to recruit 12 public schools, which we select randomly from the complete list of coeducational municipal schools from Tartumaa county, Estonia (i.e., three schools randomly allocated to each of the four study groups). Invitation letters will be sent to randomly selected schools. After confirmation, Grade 6–7 students, their parents and their PE teachers will be recruited to participate in the study. Data is collected only from participants who have agreed to participate in the study. At least one of the parents of the children will be asked to participate via eKool or Stuudium, online applications for schools in Estonia that connect teachers, parents, and children. These applications allow access to study materials, information about academic progress, and messaging in an online environment.

### 2.4. Ethical Considerations, Consent, and Permissions

The research project will be conducted in accordance with the Declaration of Helsinki and the guidelines of Good Clinical Practice. Ethical approval for the study was obtained from the Research Ethics Committee of the University of Tartu (code: 327/T-4, 19.10.2020). Important protocol modifications will be coordinated with the Research Ethics Committee of the University of Tartu.

First, the authorities of randomly selected public schools will be contacted requesting permission to conduct the study. The school authorities will be presented with the study information, including the explanation that participation is voluntary, and that withdrawal does not entail any negative consequences. In case of refusal, the next public school is contacted. After obtaining permission from the school authorities, the invitation including the study information and informed consent form will be sent to the PE teachers at the school. After obtaining consent from PE teachers, the study information and informed consent forms will be distributed to the parents of all eligible Grade 6 students. As parents are invited to take part in the study also as participants, a separate consent form will be provided to them.

The study information for the participants contains an explanation of the purpose and procedures of the study, the expected duration, and potential benefits and risks. The research does not harm the subjects mentally nor physically. Invasive research methods will not be used. The participants will be informed that the participation is entirely voluntary, the data will be handled confidentially, and they can withdraw from the study at any time without any negative consequences except forgoing the potential benefits of taking part in the intervention program. It will be affirmed that the anonymity of the participants will be guaranteed while presenting the data at conferences, publishing findings in peer-reviewed publications and in articles directed to the wider public.

As responses to questionnaires are confidential and anonymous, a personal code is created for each participant, to match the responses to questionnaires in various data points. The personal code for students will be based on the following characteristics: (i) participant’s initials; (ii) gender (B, G); (iii) class number (e.g., 6A, 6B); (iv) school name abbreviation; (v) three random digits. The personal code for parents is created by adding the extension “_par” to the end of the student’s code.

The consent forms will be distributed to participants by research team members who will answer all questions about the conduct of the study.

### 2.5. Web-Based Need-Supportive Intervention Programs

The PE teachers and parents in the experimental conditions will have access to learning platforms (i.e., Moodle) developed for this project. Although similar in structure, separate context-modified Moodle sites for PE teachers and parents will be developed. The websites guide participants through the six-week training program. The training program will be developed specifically for this project but is informed by previous autonomy-supportive intervention programs [22,29,32]. The program aims to familiarize participants with techniques directed at promoting children’s autonomous motivation towards out-of-school PA. All the training material will be presented to the participants via short video lectures. The program will begin with a brief overview of the aims and procedures of the training. The underlying theory of the training (i.e., self-determination theory) will be introduced with focus on the key components–the psychological needs for autonomy, competence, and relatedness, and their interrelationship with qualitatively different forms of motivation.

The main content of the training program includes introducing PE teachers and parents with a series of motivation and behavioral change techniques (MBCTs) recently identified by Teixeira and colleagues [20] as promising strategies to satisfy basic psychological needs in various domains, including PA. Specifically, the MBCTs cover autonomy-support techniques (e.g., providing choice, using non-controlling informational language), competence-support techniques (e.g., offering constructive, clear and relevant feedback, clarifying expectations), and relatedness-support techniques (e.g., showing unconditional regard, using empathic listening). Participants will be provided with educational units (session summaries, presentation slides) and short videos covering MBCTs adapted to school and home conditions, respectively. Following each week’s training material, participants will be asked to complete a short multiple-choice test to ensure they understood the materials. At the end of the program, PE teachers and parents will be asked to complete a comprehensive test based on all the study materials.

Following the six-week training period, PE teachers and parents will be encouraged to apply the learned MBCTs during the one-month implementation period.

## 3. Procedures

### 3.1. Outcome Measures for Students

#### 3.1.1. Behavioral Measure

The behavioral outcome measure is the students’ participation in out-of-school moderate-to-vigorous PA (MVPA) measured self-report questionnaires pre-trial, post-trial, one- and six-month data collection points. MVPA was chosen because it has been recognized as having the most health benefits (e.g., better mental health, better cardio-respiratory fitness, and less fat gain) among adolescents [33]. Students reported their PA behavior by two measures. First, an adapted version of Godin and Shepherd’s [34] leisure time exercise questionnaire was used. Previous studies have demonstrated this measure to be reliable and valid [27,35,36,37]. Secondly, the short form of the International Physical Activity Questionnaire (IPAQ) was used [38], modified to explicitly refer to leisure-time PA. Previous studies have demonstrated this measure to be reliable and valid [39,40].

#### 3.1.2. Psychological Measures

Perceived need-supportive behaviors from PE teachers and parents were measured by a battery of self-reported measures of psychological variables based on the TCM. Firstly, the modified version of the need support scale was used [41]. Previous studies have demonstrated this measure to be reliable and valid [42]. Secondly, autonomous and controlled forms of motivation in PE and out-of-school PA contexts were measured using a modified version of the Perceived Locus of Causality Questionnaire [43]. Previous studies have demonstrated this measure to be reliable and valid [35,44,45]. Thirdly, attitudes, subjective norms, perceived behavioral control, and intentions for out-of-school PA were measured using the scales developed according to the recommended guidelines [46]. Previous studies have demonstrated this measure to be reliable and valid [12,27,45].

### 3.2. Outcome Measures for Parents and Teachers

For the teachers’ and parents’ self-reports of their provision of need-support for students’ autonomy, competence, and relatedness, an adapted version of the need support scale will be used [41]. Previous studies have demonstrated this measure to be reliable and valid [42]. Furthermore, parents also reported their PA behavior by the same two measures as students (see Section 3.1.1).

### 3.3. Data Management and Analysis

Physical questionnaires will be stored in a locked cupboard in the Institute of Sport Sciences and Physiotherapy. The data from the questionnaires will be transferred to electronic format and stored on the University of Tartu server. Members of the research team (P.-R.M., H.T., A.K.) will have access to the final data set.

The data analysis will be conducted using the SPSS Version 23.0 statistical package. Randomization check will be conducted to examine the baseline differences between study groups by using the series of analysis of variance (ANOVA) and the chi-square test. An attrition check to examine the differences between those who remain in the study and those who are lost to follow-up will be conducted using the independent samples *t*-test and the chi-square test. Repeated-measures ANOVA will be used to test the effects of the intervention condition and time on the dependent variables.

To evaluate the influence of missing values, the effects of the intervention on children’s out-of-school MVPA will be analyzed using both complete-case and intent-to-treat with last observation carried forward (LOCF) methods.

## 4. Expected Results

As physical inactivity is on the rise among children and adolescents, it is crucial to find effective and cost-effective tools to at least slow down the decline in PA levels. With this project, we aim to develop web-based intervention programs for both PE teachers and parents to train them in need-supportive behaviors to support adolescents’ PA. We will evaluate the efficacy of the unique and combined effects of these programs on adolescents’ motivation towards and actual participation in out-of-school objectively measured PA. We have adopted a web-based format for delivering the need-supportive interventions to PE teachers and parents, because this enables us to reach larger audiences. During the implementation period, we plan to engage teachers and parents in forum discussions to internalize the behaviors and hence maximize the effectiveness of the interventions.

The intervention will make a unique contribution to knowledge in three areas: (i) it will test the unique and combined effects of theory-based interventions delivered via PE teachers and parents to increase students’ out-of-school PA; (ii) it will adopt an entirely online approach to evaluate the effectiveness of the interventions; (iii) it will evaluate the long-term influence of the intervention on students’ out-of-school PA through one- and six-month follow-up measurements.

A possible limitation of our project is recruiting a sufficient number of parents to take part in the intervention. Unpublished work in our institute suggests that recruiting parents is not very efficient when approached via children. We plan to add additional methods to contact parents directly (e.g., parents’ meetings in schools and parents’ groups in social networks). The benefits of participating in this project will be clearly emphasized in the study information sheet sent to parents. We will also consider providing incentives (e.g., gift cards) to encourage parents to take part in the study.

Girls tend to be more willing to participate in studies. For example, in the study of Kalajas-Tilga and colleagues [7] the participants were 68% female. Hence, we have the challenge to motivate more boys to take part in the project. For example, we will include a male member of the research group to introduce the study to possible participants. It is also very important to make it clear that participating is their own decision, not pressure from teachers or parents.

There is also the possibility that we might have difficulties reaching participants in schools due to a pandemic. In case of a lockdown, we will provide a link to an electronic questionnaire for the participants. To minimize the non-response to the online questionnaire, reminders will be sent to students through the e-School or Stuudium online environments.

It is important to develop alternative contact-free solutions to support PA. Web-based programs are cost-effective, convenient for the user and allow self-paced and contact-free learning. On the practical side, the results of the study can be used to inform future educational programs for both PE teachers and parents to teach them how to be more need-supportive in their communication with children. Due to the online approach, the intervention is also easily adaptable to other countries. Hence, if this project proves to be effective, a highly valuable web-based solution would be available for PE teachers and parents on an international level helping boost adolescents’ PA levels. In the future, the project can be expanded to include diverse age groups, and the content of the intervention can be adapted to allow students to become more need-supportive of their peers.

## Data Availability

Not applicable. Data from the experimental study will be available in Open Science Framework (OSF) data repository.

## Abbreviations

MBCT—motivation and behavioral change technique; MVPA—moderate-to-vigorous physical activity; PA—physical activity; PE—physical education; TCM—trans-contextual model of motivation; WHO—World Health Organization.
